# Winter Is Coming: Integrative Analysis of Cold Acclimation in a Freeze Tolerant Frog

**DOI:** 10.1093/iob/obaf008

**Published:** 2025-02-27

**Authors:** E E Yokum, D L Goldstein, C M Krane

**Affiliations:** Department of Biology, University of Dayton, Dayton, OH 45469, USA; Department of Biological Sciences, Wright State University, Dayton, OH 45435, USA; Department of Biology, University of Dayton, Dayton, OH 45469, USA

## Abstract

Cope's gray treefrog *Dryophytes chrysoscelis* is a seasonally freeze tolerant anuran that undergoes a preparatory period of cold acclimation in order to survive repeated freezing and thawing each winter. The mechanisms that enable freeze tolerance in this species are not entirely understood and the ecophysiological cues that regulate cold acclimation are unstudied. In the present study, we describe aspects of behavior, morphology, and physiology of frogs progressing through cold acclimation, using a previously established protocol of changing light and temperature that successfully induces the capacity to survive whole body freezing and thawing. Wild-caught males were captured in July in southwest Ohio, and behavioral, morphological, and physiological variables were compared beginning in August among frogs maintained under constant (“warm”) environmental conditions (22°C, 12:12 light: dark) and those undergoing cold acclimation (October through December) to 5°C, 8:16 light: dark. During cold acclimation frogs ceased eating, heart rate (HR) was reduced, body mass decreased, and the righting response and toe pinch reflexes were slower and less coordinated. Some of these variables also changed in animals maintained under constant, warm, and environmental conditions during the same period: feeding decreased, HR decreased, body mass increased, and dorsal skin color shifted from green to brown. However, these warm frogs began to reverse those changes and increased feeding and HR in late January. These data indicate that behavior, morphology, and physiology in *D. chrysoscelis* are subject to seasonal variations that are augmented by cold acclimation (i.e., a reduction in photoperiod and environmental temperature). Freeze competence derived from these events may be affected by volatile climates and seasonal warming.

## Spanish

La rana arbórea de Cope, *Dryophytes chrysoscelis*, es una rana tolerante a la congelación estacional que sigue por un período preparatorio de aclimatación al frío para sobrevivir a la congelación y la descongelación repetidas cada invierno. Los mecanismos que permiten la tolerancia al congelamiento en esta especie no se comprenden completamente y las señales ecofisiológicas que regulan la aclimatación al frío no han sido estudiadas. En el presente estudio, describimos aspectos del comportamiento, la morfología y la fisiología de las ranas que pasan por la aclimatación al frío, utilizando un protocolo previamente establecido de cambios en la luz y la temperatura que induce con éxito la capacidad de sobrevivir al congelamiento y descongelamiento corporal. Las ranas macho fueron atrapados en la naturaleza en julio en el suroeste de Ohio, y se compararon las variables conductuales, morfológicas y fisiológicas comenzando en agosto entre las ranas mantenidas en condiciones ambientales constantes (“cálidos”) (22°C, 12:12 claro: oscuro) y aquellas que pasaban por la aclimatación al frío (de octubre a diciembre) a 5°C, con un fotoperíodo de 8:16 claro: oscuro. Durante la aclimatación al frío, las ranas dejaron de comer, la frecuencia cardíaca (FC) se redujo, la masa corporal disminuyó y el reflejo de enderezamiento y el reflejo de pellizco de los dedos del pie fueron más lentos y menos coordinados. Algunas de estas variables también cambiaron en los animales mantenidos en condiciones ambientales cálidas y constantes durante el mismo período: la alimentación disminuyó, la FC redujo, la masa corporal aumentó y el color de la piel dorsal cambió de verde a marrón. Sin embargo, estas ranas en condiciones cálidas comenzaron a revertir esos cambios e incrementaron la alimentación y la FC a finales de enero. Estos datos indican que el comportamiento, la morfología y la fisiología de *D. chrysoscelis* están afectados a variaciones estacionales que se aumentan con la aclimatación al frío (una reducción en el fotoperíodo y la temperatura ambiental). La habilidad para sobrevivir el congelamiento atribuido a estos eventos en el frio puede ser afectada por climas volátiles y el calentamiento estacional.

## Introduction

Cope's gray treefrog *Dryophytes* (*Hyla*) *chrysoscelis* is a freeze tolerant anuran that relies on a system of cryoprotectants including glycerol, glucose, and urea to regulate cellular volume and maintain cell integrity during freezing and thawing each winter (Layne 1999; Layne and Jones 2001; do Amaral et al. 2018). Important biochemical and physiological events, including cryoprotectant synthesis (Layne and Jones 2001; Zimmerman et al. 2007), remodeling of the hepatic transcriptome (do Amaral et al. 2020), and enhanced membrane permeability to cryoprotectants (Mutyam et al. 2011) occur during cold acclimation in preparation for freezing. However, little is known about the organismal responses to cold acclimation and the potential contribution of seasonal or circannual programming that might occur in advance of freezing and thawing.

In freeze tolerant anurans like *D. chrysoscelis*, its sister species *Dryophytes* (*Hyla*) *versicolor*, and the wood frog *Lithobates sylvaticus* (*Rana sylvatica*), freeze tolerance, and the response to freezing conditions are most robust in fall and winter (Layne and Lee 1989; Costanzo et al. 2014; Rosendale et al. 2015). In summer, *D. versicolor* is able to withstand freezing temperatures for only 9 h and tolerated the freezing of up to 52% of body water, whereas winter frogs survived freezing for at least 45 h with reduced freezing of body water relative to summer animals (Layne and Lee 1989). Additionally, in *D. chrysoscelis*, red blood cells from cold acclimated frogs are inherently more freeze tolerant than red blood cells from warm frogs (Geiss et al. 2019), thermal breadth (i.e., thermal maxima and minima) varies throughout the year (Litmer and Murray 2020), and glycogen stores are elevated in cold acclimated frogs (do Amaral et al. 2018; Yokum et al. 2023b).

A myriad of important physiological events occur during cold acclimation, but the seasonal progression of those morphological and behavioral changes that precede and likely enable freeze tolerance has not been documented. In our laboratory, we developed a protocol for cold acclimation that successfully induces the ability for gray tree frogs to survive repeated freezing and thawing. In the process of implementing that protocol, we observed a number of whole-animal traits that appeared to change during the course of acclimation. Therefore, the overarching objective of this study was to examine those variables more systematically and quantitatively throughout the course of a stepwise cold acclimation protocol where temperature and photoperiod were reduced from 22°C (12:12 light:dark) to 5°C (8:16 light:dark). We hypothesized that organismal behavior, morphology, and physiology would change in parallel with biochemical hypometabolism during cold acclimation, which aids in organismal freeze tolerance in *D. chrysoscelis.*

## Materials and methods

### Wild animal collection

Wild *D. chrysoscelis* males were captured near water sources in Warren County, OH (39.500618, −84.053619) on July 12, 2021 (*N* = 33) and July 19, 2021 (*N* = 12). Animals were transported to the vivarium at the University of Dayton where they were housed and maintained as previously established for freeze tolerance studies (Goldstein et al. 2010; Mutyam et al. 2011; do Amaral et al. 2018; Geiss et al. 2019; Yokum et al. 2023b). Frogs were housed individually in standard, translucent mouse cages measuring 18.4 cm (width) × 29.2 cm (length) × 22.2 cm (height). Frogs were provided access to RO water ad libitum in the bottom of the cage and a river rock for enrichment. Frogs were housed side by side in a standard cage rack with 4–8 frog cages per row, for which their position on each shelf was randomly assigned. Frogs were offered 3/8 inch live crickets dusted with Fluker's Repta Calcium powder thrice weekly to satiation. Immediately following capture and for the following 13–14 weeks, all frogs were maintained under constant environmental conditions at 22°C with 12 h light daily (12:12). Animal cages were rotated weekly to reduce variation induced by light and thermal gradients within vivarium facilities. Animal husbandry and experimental protocols were approved by the IACUC at the University of Dayton (protocol #020-02).

### Experimental design

Behavioral, morphological, and physiological variables were observed at weekly intervals in wild-caught frogs from the time of capture in July 2021 throughout the course of cold acclimation in the autumn season (October–December 2021) and into the early winter season for one experimental group (January 2022) (Table 1, Table S1). Observations were designated in weeks relative to the start of the cold acclimation protocol (Week 0). Captured animals (“wild”) were fully assessed within 3 days of capture (*N* = 45) and sampled at regular intervals thereafter (referred to as weeks −13 to −1) (Table 1, Table S1). After the start of the cold acclimation protocol (Week 0), frogs were sampled at intervals pertaining to a reduction in environmental temperature or photoperiod: Week 2 (15°C, 10:14 light:dark), Week 3 (12°C, 9.5:14.5 light:dark), Week 5 (7.5°C, 8.5:15.5 light:dark), and Week 8 (5°C, 8:16 light:dark) (Table 1, Table S1). Cold frogs were assessed at each interval after being held at each temperature and photoperiod for 6 days, with the exception of the final cold acclimation assessment at Week 8 when frogs were held at the final temperature and photoperiod for 2 weeks prior to assessment. A group of frogs were maintained under “warm” conditions (22°C, 12:12 light:dark) throughout the cold acclimation protocol and were additionally assessed 14 weeks after the start of the cold acclimation protocol (Week 14) (Table 1). Cold frogs were unable to be assessed at this final time (Week 14) due to interference with freeze-thaw protocols (not part of this study).

**Table 1:**
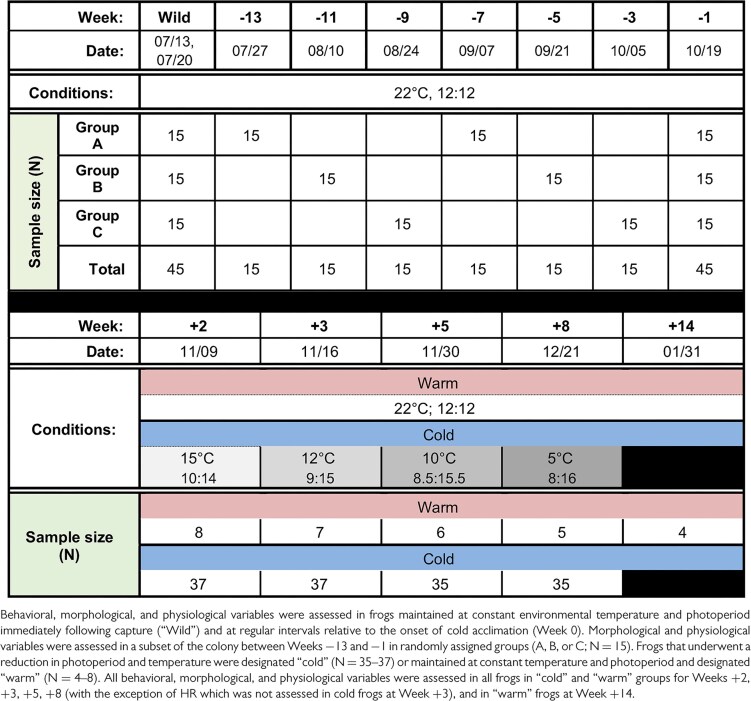
Experimental Design.

Utilizing a repeated measures design, the “wild” animal colony (*N* = 45) was randomly assigned to one of three groups (A–C) for sampling purposes only. According to group assignments, morphological and physiological variables were assessed twice between Week -13 and Week −3 at 6-week intervals so that one group was assessed every 2 weeks (*N* = 15) (Table 1, Table S1). Feeding behavior was assessed in the full colony at all-time points. One week before cold acclimation (Week −1), the entire animal colony was sampled to establish a baseline for all individuals (*N* = 45). Prior to the start of cold acclimation, the entire animal colony (groups A–C; *N* = 45) was randomly assigned to either cold (*N* = 35–37) or warm (*N* = 4–8) groups. Throughout cold acclimation, the entire cold and warm cohorts were assessed at all-time points (Table 1), with the exception of heart rate (HR) data, which were not collected on Week 3. Sample sizes were reduced in warm and cold groups throughout the course of the experiment secondary to animal mortality. Of note, frogs were collected while calling and were presumed to be male. Two frogs were later identified as female, which were not excluded from data given that responses did not vary from the rest of the cohort.

Morphological and physiological assessments were performed in the following order: photograph of dorsal surface (noncontact), measurement of HR (noncontact), body mass (manual transfer to balance), toe pinch reflex (manual transfer to flat surface and pinch with blunt forceps), and then righting response (manual inversion). All assessments were performed between 0800 and 1100 EST.

### Cold acclimation

Following capture in July, frogs were initially maintained under constant environmental conditions at 22°C with 12 h daylight (12:12, relative humidity ranged from 54–58%) from Week −14 to Week −1 (Table 1, Table S1). Beginning in October (Week 0), 37 frogs from the original cohort of 45 were randomly assigned to the cold acclimation (“cold”) protocol, hereafter used to refer to animals that underwent simultaneous changes in temperature and photoperiod. A stepwise cold acclimation protocol was implemented over the course of 8 weeks in which the environmental temperature and photoperiod were gradually reduced to 5°C with 8 h daylight (8:16, relative humidity ranged from 69–73%) consistent with previously reported cold acclimation and freeze tolerance studies (Table 1) (Zimmerman et al. 2007; Goldstein et al. 2010; Mutyam et al. 2011; do Amaral et al. 2018; Yokum et al. 2023b). With each change in environmental conditions during cold acclimation, the photoperiod was adjusted immediately, and the environmental temperature was reduced by a rate of −1°C per day. In week 4 of the cold acclimation protocol, frogs were transferred from standard mouse cages to 1-quart, vented containers and provided access to RO water in plastic weigh boats. For animals in all conditions, a remote WIFI temperature and humidity monitor was used to record environmental conditions in real time to the nearest 0.1°C.

### Behavioral analysis

Animal behavior was assessed by recording the number of crickets eaten to satiation weekly. Frogs were offered a maximum of five crickets (standard 3/8 inch size) thrice weekly, consistent with established feeding schedules and protocols in previous studies (Zimmerman et al. 2007; Goldstein et al. 2010; Mutyam et al. 2011; do Amaral et al. 2018; Yokum et al. 2023b). The number of crickets offered to and eaten by each frog was controlled and documented by aliquoting crickets for each frog in 50 mL conical tubes. Crickets were placed on a dry surface of the frog cage and uneaten crickets were removed 24 h after feeding. Frogs were initially offered 3 crickets in the vivarium following capture (based on previous husbandry records). The number of crickets offered to each frog was adjusted between each feeding based on the number of uneaten crickets left by each frog. Frogs that left uneaten crickets after 24 h were offered one less cricket at the next feeding to minimize waste. Frogs that ate all offered crickets were offered one more cricket at the next feeding (for a maximum of five total crickets per feeding). For frogs that voluntarily ceased eating, 1 cricket was offered at each feeding for up to 3 weeks before crickets were no longer offered (for frogs undergoing cold acclimation only).

### Morphological analysis

Total body weight was assessed by gently placing frogs on a small weigh boat and recording mass to the nearest 0.1 g. Relative changes in dorsal coloration were determined by digital photography using a 12-megapixel digital camera. Briefly, the cage lid was removed, and the dorsal surface of each frog was photographed from a distance of approximately 10–15 cm without disturbing the frog's position in the cage. Dorsal color was quantified by determining the relative %green (of total red, blue, and green pixilation) in each photograph as previously described using ImageJ (Touchon and Warkentin 2008; Yokum et al. 2023a).

### Physiological analysis

HR was recorded in bpm in animals both as a general indicator of stress (Cabanac and Cabanac 2000; Jacobson et al. 2021) and as an estimate of standard metabolic rate (SMR) (Steyermark et al. 2005). HR was measured in *D. chrysoscelis* using a Fitbit Versa device (Fitbit Inc, San Francisco, CA, USA), which uses photoplethysmography to detect volume changes in capillaries. The accuracy of measuring HR using this device was confirmed by simultaneous axillary palpation for animals in the following positions: frog held in hand with device directly on dorsal surface, frog held in hand with device directly on ventral surface, frog held in hand with device held approximately 2 cm from dorsal surface, and frog held in hand with ventral surface against cage container with device held directly against (transparent) cage surface. After determining the accuracy of these positions, HR was primarily measured either directly through the cage wall (on frog's ventral surface) or by holding the device just above the dorsal surface to minimize disturbance. HR was recorded within 5–10 s if readings did not vary by more than 5 bpm during that time (for which the median HR was recorded). For inconsistent readings within the initial 10 s period, animals were permitted to rest for at least 1 h before repeating measurements.

The limb withdrawal reflex (i.e., toe pinch reflex) and the righting response are common indicators of anesthetic efficacy in anurans and were evaluated in this study as a measure of nociception (toe pinch) and general neuromuscular integrity (righting response) (Layne and Kefauver 1997; Koustubhan et al. 2013; Zec et al. 2015; Cortes et al. 2016; Medler 2019). The toe pinch reflex was observed by gently placing frogs on a flat surface and pinching one of the middle toes on the frog's right hind limb with medium pressure for approximately 0.5 s. The relative speed and coordination of the frog's response was evaluated on a scale that incorporated the presence, speed, and coordination of limb withdrawal (Table 2). The righting response was observed by gently inverting the animal onto its dorsal surface and allowing the frog to recover its upright position. Responses were also evaluated on a scale that incorporated the presence, speed, and coordination of the response (Table 2). The assessment of the toe pinch reflex and the righting response were performed and scored by the same researcher to ensure consistency.

**Table 2:** Evaluation and scoring of the toe pinch reflex and righting response index.

Score	Toe pinch reflex	Righting response index
	Rapid, complete limb withdrawal	Coordinated, rapid response
**1**		
	Delayed, complete limb withdrawal	Uncoordinated, delayed, righting in <5s
**2**		
	Incomplete limb withdrawal	Uncoordinated, delayed, righting in >5s
**3**		
	Small movement or twitch only	Small movement or twitch only
**4**		
**5**	No response	No response

The toe pinch (limb withdrawal) reflex and the righting response were evaluated in individual frogs. The relative speed and coordination of each respective reflex or response was scored on a scale ranging from 1 (rapid, coordinated response) to 5 (absent response).

### Data visualization and statistical analysis

All data were initially plotted for recently captured frogs (“wild”), in frogs maintained at constant temperature and photoperiod in the weeks leading up to cold acclimation (week −13 through week −1), for cold frogs undergoing cold acclimation (Week 0 through Week 8), and for warm frogs maintained at constant temperature and photoperiod during the cold acclimation period (Week 0 through Week 14). Given the complex nature of the experimental design, variation in sample sizes throughout the experiment, and data type, specific time points of interest were selected based on scientific merit. Significant differences among weeks of interest (“wild,” Weeks: −11, −1, +2 (warm and cold), +8 (warm and cold), +14 (warm)) were determined using a repeated measures mixed-effects model with a fixed column effect and Tukey's post-hoc test to evaluate pairwise comparisons. Significance was accepted at *P* < 0.05. All data are presented as mean ± standard deviation. All statistical analyses were performed in GraphPad Prism 10.0.0.

## Results

### Behavioral analysis

**Fig. 1:**
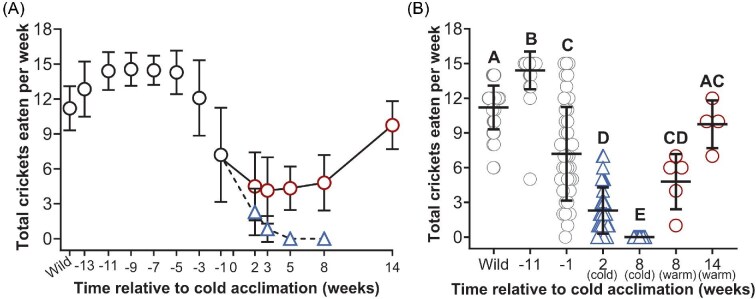
Total crickets eaten weekly to satiation in wild-caught frogs, frogs maintained under constant environmental conditions, and in cold-acclimated frogs. The mean number of crickets eaten per week are indicated in frogs maintained under constant environmental conditions (22°C, 12 h daylight) immediately following capture (wild; *N* = 45) and between Weeks −13 and −1 (*N* = 45) (black circles) (A). Beginning in Week 0, the mean number of crickets eaten per week is indicated in cold (blue triangle, dashed line; *N* = 35–37) and warm frogs (red circle, solid line; *N* = 4–8) (A). The number of crickets eaten per week was compared among frogs at biologically relevant time points: in wild-caught frogs following capture (wild; *N* = 45), in frogs eleven weeks before the start of cold acclimation (Week −11; *N* = 45), in frogs 1 week before the start of cold acclimation (Week −1; *N* = 45), in cold frogs 2 weeks after beginning cold acclimation (Week 2; *N* = 37), in cold frogs after 8 weeks of cold acclimation (Week 8; *N* = 35), in warm frogs at Week 8 (*N* = 5), and in warm frogs at Week 14 (*N* = 4) (B). Significant differences between groups are indicated by letter assignments (*P* < 0.05). All data are mean ± SD.

Feeding behavior was quantified by totaling the weekly number of crickets eaten to satiation per frog (Fig. 1A). The mean number of crickets eaten per week varied among compared time points and between warm and cold frogs [*F*_(6,165)_ = 191.7; *P* < 0.0001) (Fig. 1B). The mean total crickets eaten per week in wild-caught animals was 11.2 ± 1.9 crickets, which statistically differed from all groups (*P* < 0.0001) except the warm group at Week 14 when frogs ate an average of 7.8 ± 4.4 crickets (*P* = 0.84) (Fig. 1B). The mean number of crickets eaten per week was greatest at Week −11, which was elevated by 1.3-fold compared to wild-caught animals, and by 1.5 to 15-fold compared to all other compared groups (*P* < 0.05) (Fig. 1B). One week prior to the onset of cold acclimation (Week −1), the number of crickets eaten per week was 7.2 ± 4.0, where the standard deviation was also greatest, and statistically differed from all other groups (*P* < 0.0001) except the warm group at Week 8 and Week 14 (*P* > 0.05). The cold mean at Week 2 was 2.3 ± 2.0 and ceased entirely by Week 8 (*P* = 0.0004). The warm mean at Week 8 was 4.8 ± 2.4 crickets which nearly doubled by Week 14 (*P* = 0.0271), and also statistically differed from the cold group at Week 8 (*P* = 0.0005), but not the cold group at Week 2 (*P* = 0.2717).

### Morphological analysis

**Fig. 2:**
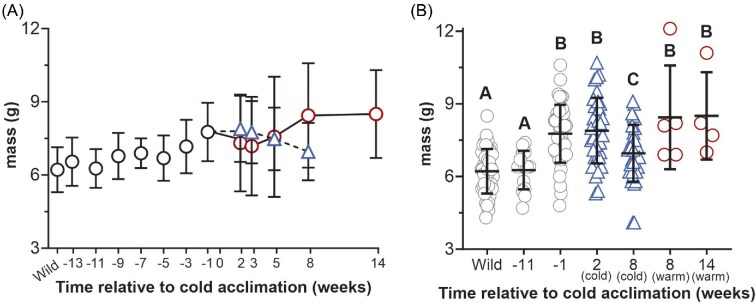
Body mass in wild-caught frogs, frogs maintained under constant environmental conditions, and in cold-acclimated frogs. The mean body mass (g) is indicated in frogs maintained under constant environmental conditions (22°C, 12 h daylight) immediately following capture (wild, *N* = 45), between Weeks −13 and −3 (*N* = 15), and 1 week before cold acclimation (Week −1; *N* = 45) (black circles) (A). Beginning in Week 0, the mean frog body mass is indicated in cold (blue triangle, dashed line; *N* = 35–37) and warm frogs (red circle, solid line; *N* = 4–8) (A). Body mass was compared among frogs at biologically relevant time points: in wild-caught frogs following capture (wild; *N* = 45), in frogs 11 weeks before the start of cold acclimation (Week −11; *N* = 15), 1 week before the start of cold acclimation (Week −1; *N* = 45), in cold frogs 2 weeks after beginning cold acclimation (Week 2; *N* = 37), in cold frogs after 8 weeks of cold acclimation (Week 8; *N* = 35), in warm frogs at Week 8 (*N* = 5), and in warm frogs at Week 14 (*N* = 4) (B). Significant differences between groups are indicated by letter assignments (*P* < 0.05). All data are mean ± SD.

Body mass was evaluated in frogs to the nearest 0.1 g (Fig. 2A). The mean body mass varied among compared time points and between warm and cold frogs [F_(6,133)_ = 39.28; *P* < 0.0001]. The average body mass in wild-caught frogs was 6.2 ± 0.9 g and did not differ from the average frog mass at Week −11 (*P* > 0.05). However, body mass was elevated by 1.3-fold in cold frogs at Week 2, 1.2-fold in cold frogs at Week 8, and by 1.4-fold in warm frogs at Weeks 8 and 14 relative to the wild-caught group (*P* < 0.05) (Fig. 2B). The colony mean one week before cold acclimation (Week −1) was 7.8 ± 1.2 g and did not differ from the cold mean at Week 2 or the warm means at Weeks 8 and 14 (*P* > 0.05) (Fig. 2B). However, during cold acclimation, body mass was reduced by 12% in frogs at Week 8 held at 5°C compared with frogs at Week 2 held at 15°C (*P* < 0.0001) (Fig. 2B). Warm frogs at Week 8 had an average body mass of 8.4 ± 2.1, which was 1.2-fold greater than the mean of the cold group at the same time point (*P* < 0.0001) (Fig. 2B). The mean body mass of the warm frog cohort at Week 8 did not statistically differ from the colony mean at Week −1 (*P* > 0.05), nor from the warm cohort at Week 14 (*P* > 0.999) (Fig. 2B).

**Fig. 3:**
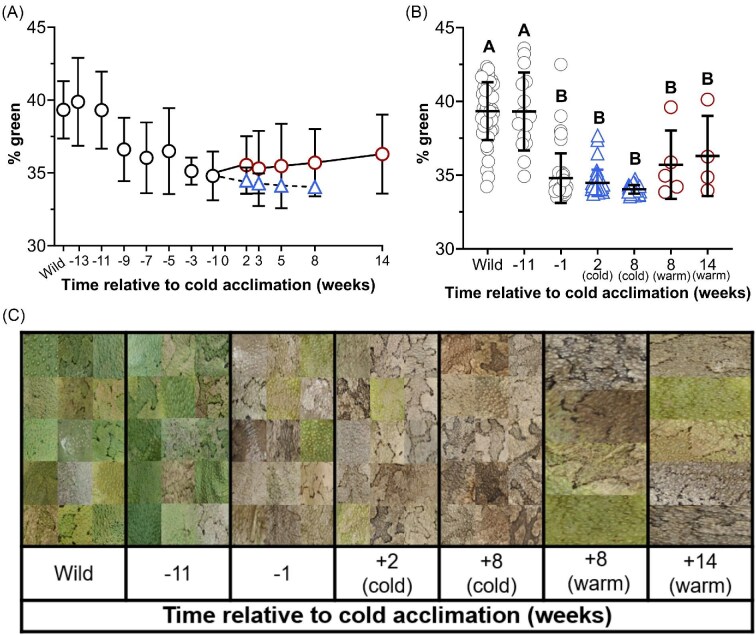
Dorsal body color in wild-caught frogs, frogs maintained under constant environmental conditions, and in cold-acclimated frogs. Dorsal body color is represented by %green of total red, blue, and green pixelation of digital photographs. The mean %green of frog dorsal color is indicated in frogs maintained under constant environmental conditions (22°C, 12 h daylight) immediately following capture (wild; *N* = 45) and between Weeks −13 and −3 (*N* = 15), and 1 week prior to beginning cold acclimation (Week −1; *N* = 45) (A). Beginning in Week 0, the mean %green of dorsal color is indicated in cold (blue triangle, dashed line; *N* = 35–37) and warm frogs (red circle, solid line; *N* = 4–8) (A). Dorsal %green was compared among frogs at biologically relevant time points: in wild-caught frogs following capture (wild; *N* = 45), in frogs 11 weeks before the start of cold acclimation (Week −11; *N* = 15), 1 week before the start of cold acclimation (Week −1; *N* = 45), in cold frogs 2 weeks after beginning cold acclimation (Week 2; *N* = 37), in cold acclimated frogs after 8 weeks of cold acclimation (Week 8; *N* = 35), in warm frogs at Week 8 (*N* = 5), and in warm frogs at Week 14 (*N* = 4) (B). Significant differences between groups are indicated by letter assignments (*P* < 0.05). All data are mean ± SD.

Dorsal color was quantified by determining the relative % green (%green) of total red, blue, and green pixels in digital images of each frog (Fig. 3A). Relative %green was quantified among groups of interest and differed between compared time points and between warm and cold frogs (F_(6,135)_ = 92.59, *P* < 0.0001) (Fig. 3B). Individual %green ranged from approximately 45% (bright green) to 33% (brown or gray) and is visually shown in representative individuals (Fig. 3C). The maximum mean %green was 39.3 ± 2.0% in wild-caught animals, which did not differ from the mean at Week −11 (*P >* 0.05) but was reduced by approximately 10% in all other compared groups (*P* < 0.05) (Fig. 3B). The dorsal %green did not differ between the colony mean at Week −1, or any warm or cold groups (*P* > 0.05). The standard deviation in the colony at Week −1 was 1.67, which was reduced by 47% and 82% in cold frogs at Week 2 and Week 8, respectively. The standard deviation of dorsal %green in cold frogs that were acclimated to 5°C (Week 8) was reduced by 87% compared with the warm frogs at the same time (Week 8).

### Physiological analysis

**Fig. 4:**
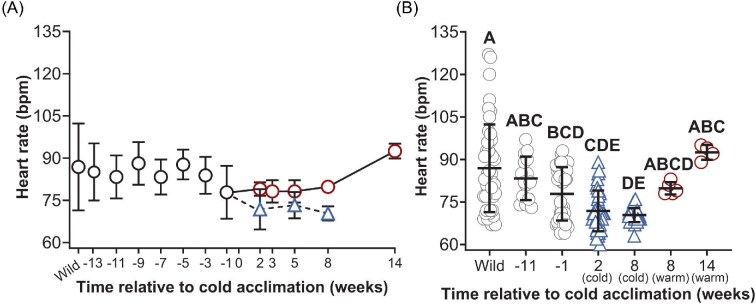
HR in wild-caught frogs, frogs maintained under constant environmental conditions, and in cold-acclimated frogs. The mean HR is indicated in frogs maintained under constant environmental conditions (22°C, 12 h daylight) immediately following capture (wild; *N* = 45), between Weeks −13 and −3 (*N* = 15), and 1 week prior to beginning cold acclimation (Week −1; *N* = 45) (black circles) (A). Beginning in Week 0, the mean HR is indicated in cold (blue triangle, dashed line, *N* = 35–37) and warm frogs (red circle, solid line; *N* = 4–8) (A). HR was compared among frogs at biologically relevant time points: in wild-caught frogs following capture (wild; *N* = 45), in frogs 11 weeks before the start of cold acclimation (Week −11; *N* = 15), 1 week before the start of cold acclimation (Week −1; *N* = 45), in cold frogs 2 weeks after beginning cold acclimation (Week 2; *N* = 37), in cold frogs following 8 weeks of cold acclimation (Week 8; *N* = 35), in warm frogs at Week 8 (*N* = 5), and in warm frogs at Week 14 (*N* = 4) (B). Significant differences between groups are indicated by letter assignments (*P* < 0.05). All data are mean ± SD.

The mean HR of frogs was reported in bpm (Fig. 4A). The mean HR varied among compared time points and between warm and cold frogs (F_(6,132)_ = 13.74; *P* < 0.0001) (Fig. 4B). The mean HR in wild-caught animals was 86.9 ± 15.5 bpm and ranged among individual frogs from 60 (Week 2, cold) to 127 (wild) bpm (Fig. 4A). The wild-caught mean was reduced by 10% at Week −1, by 17% in cold frogs at Week 2, and by 19% in cold frogs at Week 8 (*P* < 0.05) but did not statistically differ from other groups (*P* > 0.05) (Fig. 4B). The standard deviation was greatest in wild-caught frogs, and was reduced by 50% in Week −11, by 39% in Week −1, by 54% in cold frogs at Week 2, and by 84% in cold frogs at Week 2 (Fig. 4B). The colony mean at Week −1 was 77.8 ± 9.4 bpm and statistically differed from the cold mean at Week 8 (5°C) (*P* < 0.05), but not the cold mean at Week 2 (15°C) (*P* > 0.05) (Fig. 4B). The cold mean at Week 2 does not differ from the cold mean at Week 8 (*P* > 0.05), though the standard deviation was reduced by 65% during this time (Fig. 4B). The warm mean at Week 8 does not differ from the warm mean at Week 14 despite trends that demonstrate 4/4 frogs increased HR by at least 8 bpm (*P* = 0.45), and only the warm mean at Week 14 statistically differs from the cold mean at Weeks 2 and 8 (Fig. 4B).

**Fig. 5:**
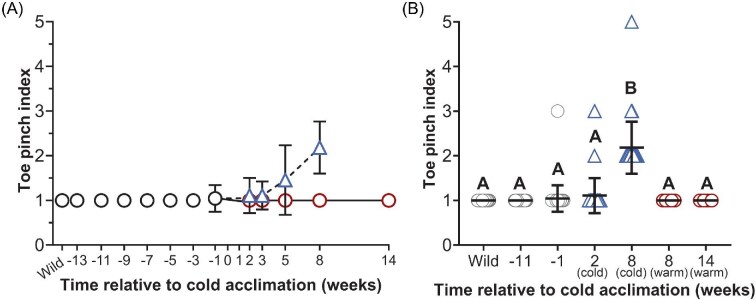
The toe pinch reflex in wild-caught frogs, frogs maintained under constant environmental conditions, and in cold-acclimated frogs. The mean toe pinch index (scored according to the criteria in Table 2) is indicated in frogs maintained under constant environmental conditions (22°C, 12 h daylight) immediately following capture (wild; *N* = 45), between Weeks −13 and −3 (*N* = 15), and 1 week prior to beginning cold acclimation (Week −1; *N* = 45) (black circles) (A). The toe pinch index was compared among frogs at biologically relevant time points: in wild-caught frogs following capture (wild; *N* = 45), in frogs 11 weeks before the start of cold acclimation (Week −11; *N* = 15), 1 week before the start of cold acclimation (Week −1; *N* = 45), in cold frogs 2 weeks after beginning cold acclimation (Week 2; *N* = 37), in cold frogs following 8 weeks of cold acclimation (Week 8; *N* = 35), in warm frogs at Week 8 (*N* = 5), and in warm frogs at Week 14 (*N* = 4) (B). Significant differences between groups are indicated by letter assignments (*P* < 0.05). All data are mean ± SD.

The toe pinch reflex was assessed by scoring an individual's response (Table 2) (Fig. 5A). The mean toe pinch index varied among time points of interest and between warm and cold frogs (F_(6,177)_ = 51.52; *P* < 0.0001) (Fig. 5B). The mean score in wild-caught frogs was 1.0 ± 0.0 (i.e., rapid, coordinated). Scores of 2, 3, and 5 (indicative of reduced coordination or an absent response) were noted in cold frogs and in the colony sampling at Week −1 (Fig. 5A and B). Though reduced coordination in the toe pinch reflex was observed in frogs at Week −1 (1.0 ± 0.3) and cold frogs in Week 2 (1.1 ± 0.4), only the cold mean at Week 8 (2.2 ± 0.6) statistically differed from any (and all) other groups (*P* < 0.0001) (Fig. 5B).

**Fig. 6:**
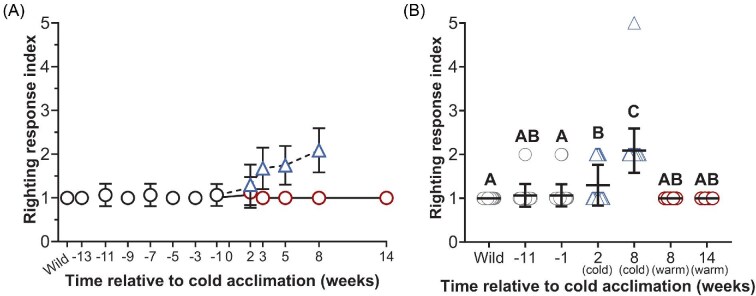
The righting response in wild-caught frogs, frogs maintained under constant environmental conditions, and in cold-acclimated frogs. The mean righting response index (scored according to the criteria in Table 2) is indicated in frogs maintained under constant environmental conditions (22°C, 12 h daylight) immediately following capture (wild; *N* = 45), between Weeks −13 and −3 (*N* = 15), and 1 week prior to beginning cold acclimation (Week −1; *N* = 45) (black circles) (A). The righting response index was compared among frogs at biologically relevant time points: in wild-caught frogs following capture (wild; *N* = 45), in frogs 11 weeks before the start of cold acclimation (Week −11; *N* = 15), 1 week before the start of cold acclimation (Week −1; *N* = 45), in cold frogs 2 weeks after beginning cold acclimation (Week 2; *N* = 37), in cold frogs following 8 weeks of cold acclimation (Week 8; *N* = 35), in warm frogs at Week 8 (*N* = 5), and in warm frogs at Week 14 (*N* = 4) (B). Significant differences between groups are indicated by letter assignments (*P* < 0.05). All data are mean ± SD.

The righting response was assessed by scoring an individual's response (Table 2) (Fig. 6A). The mean righting response index varied among time points of interest and between warm and cold frogs [F_(6,135)_ = 44.17; *P* < 0.0001] (Fig. 6B). The mean score in wild-caught frogs was 1.0 ± 0.0 (i.e., rapid, coordinated) and scores of 2 and 5 (i.e., reduced coordination or absent righting response) were recorded in cold frogs and among the colony at Week −1 (Fig. 6A). The wild-caught mean did not differ from the colony mean at Week −11 or Week −1 but was elevated by 1.3-fold and 2.1-fold in cold frogs at Weeks 2 and 8, respectively (*P* < 0.0001) (Fig. 6B). The cold mean at Week 2 (15°C) differed from the wild-caught mean, the colony mean at Week −1, and the cold mean at Week 8 (*P* < 0.05), but not the colony mean at Week −11 or the warm means at Weeks 8 and 14 (*P* > 0.05) (Fig. 6B). In contrast, the cold mean at Week 8 statistically differed from all other compared groups (*P* < 0.0001) (Fig. 6B).

## Discussion

Previous studies of freeze tolerance in *D. chrysoscelis* have documented changes to cellular and organ function and have focused on the end points of the acclimation process (warm- vs. cold-acclimated states; e.g., Yokum et al. 2023b). This study is the first to systematically document behavioral, morphological, and physiological changes in a freeze tolerant animal through the progression of cold acclimation where both temperature and photoperiod were reduced, consistent with natural seasonal fluctuation. We observed responses to the initial decrease in temperature and photoperiod during cold acclimation as well as additional changes that occurred only in animals fully acclimated to 5°C. We also documented clear phenotypes associated with the wild-caught condition and in frogs maintained under constant environmental conditions. The concurrent changes in behavior, morphology, and physiology indicate underlying seasonal rhythms that are further accentuated by environmental cues. Together, these contribute to cold acclimation and ultimately freeze tolerance in *D. chrysoscelis.*

### Underlying physiological mechanisms during cold acclimation

Cold acclimation is considered critical to impart freeze competence in *D. chrysoscelis* and is commonly associated with metabolic rate depression (do Amaral et al. 2020; K. B. Storey and Storey 2017), cryoprotectant accumulation (Irwin and Lee 2003; Zimmerman et al. 2007), and other cellular responses. The organismal changes that we observed at the onset, throughout, and at the completion of the cold acclimation period were consistent with a reduced metabolic rate and lower activity levels. As cold acclimation progressed, frogs initially ate less, then ceased eating entirely, accompanied by lowered HRs. Body mass was initially unaffected by a reduction in photoperiod and temperature but was ultimately reduced in fully cold acclimated frogs by approximately 12%. Of note, though some frogs had stopped eating within the first 2 weeks of cold acclimation, and most had stopped eating within the first 3 weeks of cold acclimation, the mean body mass of frogs was maintained throughout at least the first 5 weeks of cold acclimation. The eventual loss in body mass likely reflects the catabolism of energy stores including hepatic glycogen and fatty acids. Though body water content did not vary between warm and cold *D. chrysoscelis* in a previous study (do Amaral et al. 2018), changes in hydration may contribute to variable body mass in this study. Carbohydrate fuel sources are particularly critical to freeze tolerant animals like *D. chrysoscelis*, which utilize glycogen in the liver and skeletal muscle for cryoprotectant synthesis during freezing (do Amaral et al. 2018; Yokum et al. 2023b). Glycogen is even further reduced with repeated freezing and thawing in *D. chrysoscelis* (Yokum et al. 2023b), suggesting that organismal survival may be limited in the event of below average temperatures, prolonged winters, or frequent temperature fluctuations that induce cycles of freezing and thawing.

The slowed and reduced coordination of the toe pinch reflex and the righting response were pronounced in cold frogs compared with warm frogs and were entirely absent in one frog that was cold acclimated to 5°C. The toe pinch reflex is indicative of nociception and follows a relatively simple, polysynaptic reflex arc (Koustubhan et al. 2013; Medler 2019), while the righting response is more complex and requires entire body coordination and movement. The simultaneous slowing of both reflexes indicates underlying neuromuscular events that potentially reflect changes in rates of synaptic transmission, membrane depolarization and repolarization, or possibly blood flow in cold acclimated animals. Both the structures and integrity of neuromuscular membranes and the rates of processes occurring in those membranes may be altered by cold acclimation. Adaptation of biological membranes to environmental fluctuation (i.e., temperature, pressure, etc.) is generally considered to be a universal feature of living things (Sinensky 1974; Hazel and Williams 1990), but delayed reflexes in cold frogs may indicate that membrane lipids in nerve and skeletal muscle tissues do not fully cold-acclimate.

### Temperature (and photoperiod) sensing during cold acclimation

All observed variables underwent change during cold acclimation, reflective of a coordinated organismal response. Compared with the warm control, in which all variables remained relatively constant during Weeks 1–8, differences observed in cold-acclimating animals indicate responses to environmental fluctuation. Though the mean dorsal color was not affected by cold, the standard deviation for this variable decreased dramatically during cold acclimation, indicating a more constrained phenotype. A similar decline in inter-individual variation was also noted in eating behavior and HR in fully cold acclimated animals relative to animals maintained in warm conditions just 1 week prior to beginning cold acclimation. These trends are reminiscent of those detected for the hepatic transcriptome, for which variation also was reduced in cold frogs (do Amaral et al. 2020). Interindividual variation in phenotype is a prominent feature of *D. chrysoscelis*, as evidenced in the wild-caught frogs in this study. The converged phenotypes that we have measured in cold frogs support the hypothesis that temperature (and photoperiod) sensing likely underlies behavioral, morphological, and physiological responses.

The timing of cold acclimation differed for the behavioral, morphological, and physiological responses that we assessed; some variables were affected by a minor reduction in temperature and photoperiod, some progressively changed throughout cold acclimation, and others only differed in frogs at the end of the cold acclimation protocol. The fastest responding variables were HR and feeding behavior, which diminished upon initial changes in temperature and photoperiod. In contrast, the standard deviation, but not the mean, of body mass and dorsal color appeared to progressively decline throughout cold acclimation, and the righting response and toe pinch reflex were largely unaffected until frogs reached an ambient temperature of 10°C (8.5h daylight). These variations in degree and timing of different behavioral, morphological, and physiological responses suggest tissue-specific sensing mechanisms and responses; cold sensing appears to be an integral part of metabolic regulation, while other functions like neuro-motor responsiveness are less sensitive.

### The role of seasonal acclimation in cold acclimation and freeze tolerance

One notable finding of this study is that behavioral, morphological, and physiological changes were observed in animals maintained under constant conditions. For example, HR was extremely variable in the first 24–72 h of captivity, but then HR and feeding rate stabilized during the subsequent 3–4 weeks. We interpret these patterns as reflecting the time required to overcome the stress of capture and introduction to a laboratory setting. To the best of our knowledge, the effects of capture have not been evaluated in a freeze tolerant vertebrate. These results indicate that without sufficient time for lab acclimation, physiological results may be confounded by a stress response associated with capture.

Even after that initial adjustment and the persistence of stable environmental conditions, organismal properties continued to change after several weeks in captivity. The mean number of crickets eaten per frog was approximately 15 per week for the first 6 weeks following adjustment to the laboratory setting (approximately Week −13). This decreased by approximately 30% several weeks before cold acclimation (Week −3) and by about 50%, 1 week before the start of cold acclimation (Week −1). Of note, a decrease in HR was observed at the same time, suggesting that metabolic changes may reflect this loss in appetite. Hypometabolism is a characteristic hallmark of cold acclimated animals (Heldmaier et al. 2004; Davies et al. 2015; J. M. Storey and Storey 1985), and has been previously observed at the transcriptional level in this species (do Amaral et al. 2020). Interestingly, while animals reduced feeding rates, their mean body mass continued to increase steadily from the time of capture to the beginning of cold acclimation. Though body water content was not affected by cold acclimation in a previous study of this species (do Amaral et al. 2018), changes in hydration likely underlie the observed increase in animal body mass, while caloric intake decreased prior to the onset of cold acclimation.

The other change that occurred in the absence of environmental cues was the browning of dorsal skin color. Frog skin color was generally green at the time of capture, which gradually converted to an overall brown color prior to the onset of cold acclimation. Dorsal color change and the conversion of green to brown skin in amphibians have generally been ascribed to regulation of body water and temperature or to predation avoidance (Edgren 1954; Nielsen and Dyck 1978; Smith et al. 2016). Of those possibilities, a connection to water balance seems most likely in our captive *D. chrysoscelis*. However, the conversion of green to brown skin in the absence of environmental change likely reflects neurohormonal shifts associated with seasonal rhythms.

Brown skin and diminished appetite and metabolism are characteristic of the cold acclimation phenotype in *D. chrysoscelis*. Remarkably, for animals that were maintained throughout the experiment in warm conditions (for a total of approximately 28 weeks in a constant environment between August and January), a “summer” phenotype returned in late January, after 10 weeks of “winter” phenotype. Between Week 8 and Week 14, frogs approximately doubled the number of crickets eaten per week, increased HR by as much as 10 bpm, and reestablished a greener dorsal coloration. These frogs also called at night in the absence of females (personal observations).

Behavioral, morphological, and physiological changes in the absence of environmental cues are not unprecedented in overwintering animals; complex behaviors are subject to seasonal and circannual rhythms in a variety of species, including amphibians, birds, insects, and mammals (Bertolucci et al. 1999; Dawson et al. 2001; Grace 2003; Bradshaw and Holzapfel 2007; Visser et al. 2010). At least some of those processes, such as diapause in insects, can be influenced by environmental factors (including climate change) on top of the underlying seasonal rhythm (Marshall et al. 2020). Likewise, we provide strong evidence that cold acclimation (including freeze tolerance) in *D. chrysoscelis* is regulated by a combination of seasonal rhythms and a reduction in temperature and photoperiod: (1) frogs undergo metabolic remodeling in the absence of environmental cues, (2) frogs exhibit premature mating behavior in the absence of females and without a reduction in temperature and photoperiod typical of a winter season, and (3) tissue-specific sensing of environmental fluctuation occurs during cold acclimation. Determining whether these seasonal changes reflect endogenous circannual rhythms would require further, longer-term study. The combinatorial and complex mechanisms that regulate seasonal acclimation, cold acclimation, and freeze tolerance in *D. chrysoscelis* collectively suggests that *D. chrysoscelis* is vulnerable to climate change and can serve as a model organism to better understand the effects of environmental variability on freeze tolerance.

### Conclusions and perspectives

*Dryophytes chrysoscelis* is a seasonally freeze tolerant species, for which acclimation to season and to cold are evidenced by changes in behavior, morphology, and physiology. While some of these changes were time (season)-dependent (i.e., they occurred in the absence of environmental perturbation), many changes were accentuated by diminished temperature and photoperiod, indicating that those cues are likely important for organismal cold acclimation and freeze tolerance. Given the complex nature of seasonal acclimation in this species, it is likely that early cooling and late warming events may interfere with the ability to survive freezing and thawing in winter. Moreover, seasonal hardiness and survival may be influenced additionally by factors other than those we manipulated (photoperiod and temperature), such as length of day to night transitions (dawn and dusk), precipitation, daily climatic variation, and tree cover (Huey and Buckley 2022). The importance of seasonal rhythms in *D. chrysoscelis* indicates that future work should consider the timing of ecophysiological studies. Warm-acclimated controls should be appropriately time matched to cold, frozen, or thawed groups. This study focused on a single freeze tolerant species *D. chrysoscelis*, but these findings are likely relevant to many freeze- and cold tolerant animal species in the northern hemisphere.

## Supplementary Material

obaf008_Supplemental_File

## Data Availability

Data and raw photographs are available from the corresponding author upon reasonable request.

## References

[bib1] Bertolucci C, Leorati M, Innocenti A, Foà A. 1999. Circannual variations of lizard circadian activity rhythms in constant darkness. Behav Ecol Sociobiol 46:200–9. 10.1007/s002650050610

[bib2] Bradshaw WE, Holzapfel CM. 2007. Evolution of animal photoperiodism. Annu Rev Ecol Evol Syst 38:1–25. 10.1146/ANNUREV.ECOLSYS.37.091305.110115

[bib3] Cabanac A, Cabanac M. 2000. Heart rate response to gentle handling of frog and lizard. Behav Processes 52:89–95. 10.1016/S0376-6357(00)00108-X 11164677

[bib4] Cortes PA, Puschel H, Acuña P, Bartheld JL, Bozinovic F. 2016. Thermal ecological physiology of native and invasive frog species: do invaders perform better? Conserv Physiol 4:1–10. 10.1093/CONPHYS/COW056 27933168 PMC5141634

[bib5] Costanzo JP, do Amaral MCF, Rosendale AJ, Lee RE. 2014. Seasonality of freeze tolerance in a subarctic population of the wood frog, *Rana sylvatica*. Int J Zool 2014:1–13. 10.1155/2014/750153

[bib6] Davies SJ, McGeoch MA, Clusella-Trullas S. 2015. Plasticity of thermal tolerance and metabolism but not water loss in an invasive reed frog. Comp Biochem Physiol A: Mol Integr Physiol 189:11–20. 10.1016/J.CBPA.2015.06.033 26164532

[bib7] Dawson A, King VM, Bentley GE, Ball GF. 2001. Photoperiodic Control of Seasonality in Birds. J Biol Rhythms 16:365–80. 10.1177/074873001129002079 11506381

[bib8] do Amaral MCF, Frisbie J, Crum RJ, Goldstein DL, Krane CM. 2020. Hepatic transcriptome of the freeze-tolerant Cope's gray treefrog, *Dryophytes chrysoscelis*: responses to cold acclimation and freezing. Bmc Genomics [Electronic Resource] 21:1–18. 10.1186/s12864-020-6602-4 PMC7069055 32164545

[bib9] do Amaral MCF, Frisbie J, Goldstein DL, Krane CM. 2018. The cryoprotectant system of Cope's gray treefrog, *Dryophytes chrysoscelis*: responses to cold acclimation, freezing, and thawing. J Comp Physiol B 188:611–21. 10.1007/s00360-018-1153-6 29550887 PMC6006228

[bib10] Edgren RA . 1954. Factors controlling color change in the tree frog, *Hyla versicolor*. Exp Biol Med 87:20–3. 10.3181/00379727-87-21272 13224664

[bib11] Geiss L, do Amaral MCF, Frisbie J, Goldstein DL, Krane CM. 2019. Postfreeze viability of erythrocytes from *Dryophytes chrysoscelis*. J Exp Zool Pt A 331:308–13. 10.1002/jez.2262 30933437

[bib12] Goldstein DL, Frisbie J, Diller A, Pandey RN, Krane CM. 2010. Glycerol uptake by erythrocytes from warm- and cold-acclimated Cope's gray treefrogs. J Comp Physiol B 180:1257–65. 10.1007/s00360-010-0496-4 20652259

[bib13] Grace MS . 2003. Timing of reproductive immigration in salamanders: roles of environmental cues and endogenous biological clocks. Herpetol Rev 34:17–20.

[bib14] Hazel JR, Williams E. 1990. The role of alterations in membrane lipid composition in enabling physiological adaptation of organisms to their physical environment. Prog Lipid Res 29:167–227. 2131463 10.1016/0163-7827(90)90002-3

[bib15] Heldmaier G, Ortmann S, Elvert R. 2004. Natural hypometabolism during hibernation and daily torpor in mammals. Respir Physiol Neurobiol 141:317–29. 10.1016/J.RESP.2004.03.014 15288602

[bib16] Huey RB, Buckley LB. 2022. Designing a seasonal acclimation study presents challenges and opportunities. Integr Org Biol 4:1–18. 10.1093/iob/obac016 PMC9175191 35692903

[bib17] Irwin J, Lee R. 2003. Geographic variation in energy storage and physiological responses to freezing in the gray treefrogs *Hyla versicolor* and H. chrysoscelis. J Exp Biol 206:2859–67. 12847129 10.1242/jeb.00500

[bib18] Jacobson C, Doss GA, Yaw TJ, Mans C, Sladky KK. 2021. Effect of manual restraint and visual security on heart rate in dyeing poison dart frogs (Dendrobates *Tinctorius azureus*) and leopard frogs (*Lithobates pipiens*). J Herpetol Med Surg 31:59–63. 10.5818/20-00021.1

[bib19] Koustubhan P, Kaplan DL, Levin M. 2013. Humane anesthesia and pain management in amphibian limb surgery of rana pipiens. Cold Spring Harb Protoc 2013:149–55. 10.1101/PDB.PROT071977 23378649 PMC3768120

[bib20] Layne JR . 1999. Freeze tolerance and cryoprotectant mobilization in the gray treefrog (*Hyla versicolor*). J Exp Zool 283:221–5. 9933936 10.1002/(sici)1097-010x(19990215)283:3<221::aid-jez1>3.0.co;2-q

[bib21] Layne JR, Jones AL. 2001. Freeze tolerance in the gray treefrog: cryoprotectant mobilization and organ dehydration. J Exp Zool 290:1–5. 10.1002/JEZ.1030 11429758

[bib22] Layne JR, Kefauver J. 1997. Freeze tolerance and postfreeze recovery in the frog *Pseudacris crucifer*. Copeia 1997:260–4.

[bib23] Layne JR, Lee RE. 1989. Seasonal variation in freeze tolerance and ice content of the tree frog *Hyla versicolor*. J Exp Zool 249:133–7. 10.1002/JEZ.1402490203 2723602

[bib24] Litmer AR, Murray CM. 2020. Critical thermal capacities of *Hyla chrysoscelis* in relation to season. J Herpeto 54:413–7. 10.1670/19-124

[bib25] Marshall KE, Gotthard KB, Williams. 2020. Evolutionary impacts of winter climate change on insects. Curr Opin Insect Sci 41:54–62. 32711362 10.1016/j.cois.2020.06.003

[bib26] Medler S . 2019. Anesthetic MS-222 eliminates nerve and muscle activity in frogs used for physiology teaching laboratories. Adv Physiol Educ 43:69–75. 10.1152/advan.00114.2018 30694709

[bib27] Mutyam V, Puccetti MV, Frisbie J, Goldstein DL, Krane CM. 2011. Dynamic regulation of aquaglyceroporin expression in erythrocyte cultures from cold- and warm-acclimated cope's gray treefrog, *Hyla chrysoscelis*. J Exp Zool 315A:424–37. 10.1002/jez.689 21656914

[bib28] Nielsen HI, Dyck J. 1978. Adaptation of the tree frog, *Hyla cinerea*, to colored backgrounds, and the role of the three chromatophore types. J Exp Zool 205:79–94. 10.1002/jez.1402050111

[bib29] Rosendale AJ, Lee RE, Costanzo JP. 2015. Seasonal variation and freezing response of glucose transporter 2 in liver of the wood frog: implications for geographic variation in freeze tolerance. J Zool 297:132–8. 10.1111/jzo.12255

[bib30] Sinensky M . 1974. Homeoviscous adaptation-A homeostatic process that regulates the viscosity of membrane lipids in *Escherichia coli*. Proc Natl Acad Sci U.S.A 71:522–5. 4360948 10.1073/pnas.71.2.522 PMC388039

[bib31] Smith KR, Cadena V, Endler JA, Kearney MR, Porter WP, Stuart-Fox D. 2016. Color change for thermoregulation versus camouflage in free-ranging lizards. Am Nat 188:668–78. 10.1086/688765 27860512

[bib32] Steyermark AC, Miamen AG, Feghahati HS, Lewno AW. 2005. Physiological and morphological correlates of among-individual variation in standard metabolic rate in the leopard frog *Rana pipiens*. J Exp Biol 208:1201–8. 10.1242/jeb.01492 15767318

[bib33] Storey JM, Storey KB. 1985. Adaptations of metabolism for freeze tolerance in the gray tree frog, *Hyla versicolor*. Can J Zool 63:49–54. 10.1139/z85-009

[bib34] Storey KB, Storey JM. 2017. Molecular physiology of freeze tolerance in vertebrates. Physiol Rev 97:623–65. 10.1152/physrev.00016.2016 28179395

[bib35] Touchon J, Warkentin K. 2008. Fish and dragonfly nymph predators induce opposite shifts in color and morphology of tadpoles. Oikos 117:634–40. 10.1111/j.0030-1299.2008.16354.x

[bib36] Visser ME, Caro SP, Oers KV, Schaper SV, Helm B. 2010. Phenology, seasonal timing and circannual rhythms: towards a unified framework. Phil Trans R Soc B 365:3113–27. 10.1098/rstb.2010.0111 20819807 PMC2981940

[bib37] Yokum EE, Goldstein DL, Krane CM. 2023a. Novel observations of “freeze resistance” and dynamic blue and green dorsal coloration in frozen and thawing *Dryophytes chrysoscelis*. J Exp Zool Pt A 339:1044–51. 10.1002/jez.2753 37661700

[bib38] Yokum EE, Wascher M, Goldstein DL, Krane CM. 2023b. Repeated freeze-thaw cycles in freeze tolerant treefrogs: novel intra-individual variation of integrative biochemical, cellular, and organismal responses. Am J Physiol-Regul, Integr Comp Physiol 324:R196–206. 10.1152/ajpregu.00211.2022 36534587

[bib39] Zec S, Clark-Price SC, Coleman DA, Mitchell MA. 2015. Loss and return of righting reflex in American green tree frogs (*Hyla cinerea*) after topical application of compounded sevoflurane or isoflurane jelly: a pilot study. J Herpetol Med Surg 24:72. 10.5818/1529-9651-24.3.72

[bib40] Zimmerman SL, Frisbie J, Goldstein DL, West J, Rivera K, Krane CM. 2007. Excretion and conservation of glycerol, and expression of aquaporins and glyceroporins, during cold acclimation in Cope's gray tree frog *Hyla chrysoscelis*. Am J Physiol-Regul, Integr Comp Physiol 292:R544–55. 10.1152/ajpregu.00434.2006 16973932

