# Supplementary material for: Winter Is Coming: Integrative Analysis of Cold Acclimation in a Freeze Tolerant Frog
Source: Integr Org Biol. 2025 Feb 27;7(1):obaf008. doi: 10.1093/iob/obaf008 (PMC11933796; doi:10.1093/iob/obaf008)
Supplement: obaf008_Supplemental_File [file obaf008_supplemental_file.pdf]

Date	Week Designation	N (crickets)	N (body mass, dorsal color, HR, toe pinch, righting response)	Notes
13 July, 20 July	Wild	45	45	Frogs collected on 12 July (N=33) were assessed in the lab on 13 July. Frogs collected on 19 July (N=12) were assessed in the lab on 20 July. All assessments of recently captured frogs were combined into a single "wild" group. All frogs maintained at 22°C (12 h daylight).
27 July	-13	45	15 (group A)	For all variables (except feeding behavior), all frogs were randomly assigned to groups A, B, or C which were sampled every 6 weeks.
10 Aug	-11	45	15 (group B)	
24 Aug	-9	45	15 (group C)	
07 Sept	-7	45	15 (group A)	
21 Sept	-5	45	15 (group B)	
05 Oct	-3	45	15 (group C)	
19 Oct	-1	45	45	Last collection point before start of cold acclimation
09 Nov	+2	37 (cold), 8 (warm)	37 (cold), 8 (warm)	Environmental temperature reduced to 15°C (10 h daylight) for cold frogs.
16 Nov	+3	37 (cold), 7 (warm)	37 (cold), 7 (warm)	Environmental temperature reduced to 12°C (9 h daylight) for cold frogs. Heart rate was not assessed in cold frogs on this date.
30 Nov	+5	35 (cold), 6 (warm)	35 (cold), 6 (warm)	Environmental temperature reduced to 10°C (8.5 h daylight) for cold frogs.
21 Dec	+8	35 (cold), 5 (warm)	35 (cold), 5 (warm)	Environmental temperature reduced to 5°C (8 h daylight) for cold frogs. Final collection point of cold-acclimated frogs
31 Jan	+14	4 (warm)	4 (warm)	

Experimental design with sampling and data collection detail. *Dryophytes* (*Hyla*) *chrysoscelis* were captured in July 2021 and transported to a laboratory setting. Behavioral (number of crickets eaten per week), morphological (dorsal color, body mass), and physiological (heart rate, toe pinch reflex, righting response) variables were assessed within 72 h of capture and throughout the experimental timeline until late January 2022.
